# Signaling to stomatal initiation and cell division

**DOI:** 10.3389/fpls.2014.00297

**Published:** 2014-06-23

**Authors:** Jie Le, Junjie Zou, Kezhen Yang, Ming Wang

**Affiliations:** Key Laboratory of Plant Molecular Physiology, Institute of Botany, Chinese Academy of SciencesBeijing, China

**Keywords:** stomata, development, signaling, cell fate, cell division

## Abstract

Stomata are two-celled valves that control epidermal pores whose opening and spacing optimizes shoot-atmosphere gas exchange. *Arabidopsis* stomatal formation involves at least one asymmetric division and one symmetric division. Stomatal formation and patterning are regulated by the frequency and placement of asymmetric divisions. This model system has already led to significant advances in developmental biology, such as the regulation of cell fate, division, differentiation, and patterning. Over the last 30 years, stomatal development has been found to be controlled by numerous intrinsic genetic and environmental factors. This mini review focuses on the signaling involved in stomatal initiation and in divisions in the cell lineage.

## INTRODUCTION

**FIGURE 1:**
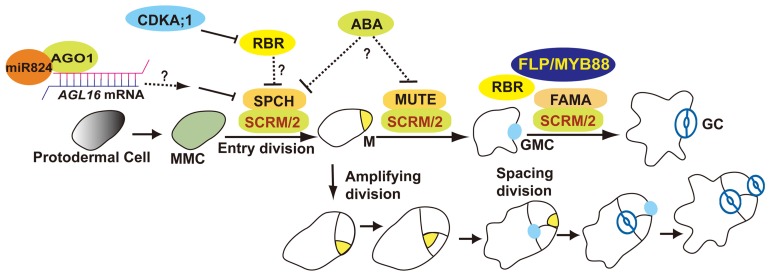
**Overview of stomatal development.** The stomatal lineage initiates from meristemoid mother cells (MMCs). MMC undergoes asymmetric entry divisions and produces a meristemoid (M) as well as a larger sister cell (termed a stomatal lineage ground cell, SLGC). Meristemoids can undergo asymmetric amplifying divisions before differentiating into a guard mother cell (GMC). SLGCs can differentiate into a pavement cells or reacquire a MMC fate and initiate asymmetric spacing divisions that produce “satellite meristemoids.” Stomatal complexes form after at least one unequal division of a stem cell, and then by a single equal division of a GMC. SPCH, MUTE, FAMA, and FLP/MYB88 are transcription factors that regulate key fate transitions during stomatal development. SCRM and SCRM2 heterodimerize with SPCH, MUTE, and FAMA to promote stomatal–lineage transitions. AGO1 is involved in an AGL16-mediated microRNA post-transcriptional regulatory pathway that blocks asymmetric spacing divisions in SLGCs. ABA might be involved in stomatal initiation and differentiation by repressing SPCH and MUTE. RBR activity is predominantly regulated by CDKA;1. RBR participates in the regulation of asymmetric as well as symmetric divisions.

Stomata formation in *Arabidopsis thaliana* involves at least one asymmetric division as well as a single symmetric division. The meristemoid mother cell (MMC) undergoes an asymmetric entry division that produces a small triangular meristemoid and a larger sister cell. The large cell, termed a stomatal lineage ground cell (SLGC), can either differentiate into a pavement cell (ubiquitous epidermal cell) or undergo asymmetric spacing divisions that produce a satellite meristemoid. Meristemoids can undergo one to three rounds of amplifying divisions before they finally differentiate into an oval-shaped guard mother cell (GMC). GMCs divide symmetrically once thus generating a pair of guard cells (GCs; **Figure 1**; Nadeau and Sack, 2002b; Bergmann and Sack, 2007).

## INTERCELLULAR SIGNALING TO STOMATAL INITIATION AND PATTERNING

Stomata are distributed and spaced throughout the plant shoot epidermis. Communication between stomata and their with neighboring epidermal cells ensures that stomata are spaced at least one cell apart (“one cell spacing” rule). The *TOO MANY MOUTHS* (*TMM*) gene was the first stomatal gene identified in *Arabidopsis* (Yang and Sack, 1995). TMM as well as three ERECTA-family (ERf) members are leucine-rich repeat (LRR) receptor-like protein and kinases. Mutations in *TMM* lead to excessive clustered stomata in leaves (Nadeau and Sack, 2002a). The loss of function of three *Arabidopsis* ERf genes, *ER*, *ERECTA-LIKE 1* (*ERL1*), and *ERECTA-LIKE 2* (*ERL2*), induces stomatal clustering (Shpak et al., 2005).

Genetic and biochemical evidence indicate that ERfs act specifically with respect to ligands and developmental stage during stomatal development. The *EPIDERMAL PATTERNING FACTOR* (*EPF*) and *EPF-LIKE* (*EPFL*) genes encode secreted cysteine-rich peptides (Torii, 2012). EPF1 and EPF2 were the first two peptides identified that are used as intercellular signals in stomatal patterning (Hara et al., 2007, 2009; Hunt and Gray, 2009). *EPF1* is expressed in late meristemoids and in GMCs in the stomatal lineage (Hara et al., 2007). *EPF2* expression is restricted to MMCs and early meristemoids, a stage earlier in stomatal development than that of *EPF1.* The loss of *EPF2* function induces excessive divisions and increased stomatal density (Hara et al., 2009; Hunt and Gray, 2009). Overexpression of *EPF2* represses *TMM* expression and blocks stomatal formation, consistent with EPF2 restricting the formation of stomatal precursors (Hunt and Gray, 2009). The loss of *EPF1* function leads to the formation stomata in contact, whereas the overexpression of *EPF1* results in few or no stomata, consistent with EPF1 regulating stomatal patterning (Hara et al., 2007). Bioactive EPF peptides have been shown to directly bind ERf receptors using biosensor chips. The *in vivo* specificities of EPFs and ERf have also been defined (Lee et al., 2012). The EPF2–ER pair prevents cells next to MMCs or Ms from acquiring a stomatal cell fate. The EPF1–ERL1 pair ensures the one-celled spacing between stomata. ER and ERL1 form homodimers or heterodimers, but TMM only forms heterodimeric receptor complexes with ERf members. Thus, stomatal lineage cell determination and patterning are precisely controlled by diverse ligand–receptor pairs (Torii, 2012).

STOMAGEN/EPFL9 is also a member of the EPF/EPFL-family of peptides that is secreted from mesophyll cells of immature leaves (Hunt et al., 2010; Kondo et al., 2010; Sugano et al., 2010). The loss *STOMAGEN/EPFL9* function using gene silencing via RNA interference resulted in a reduction in stomatal density (Hunt et al., 2010; Sugano et al., 2010). In contrast to role of *EPF1* and *EPF2*, the ectopic overexpression of *STOMAGEN* or the application of synthetic STOMAGEN peptides induce the formation of clusters containing numerous stomata in contact (Kondo et al., 2010; Sugano et al., 2010).

**FIGURE 2:**
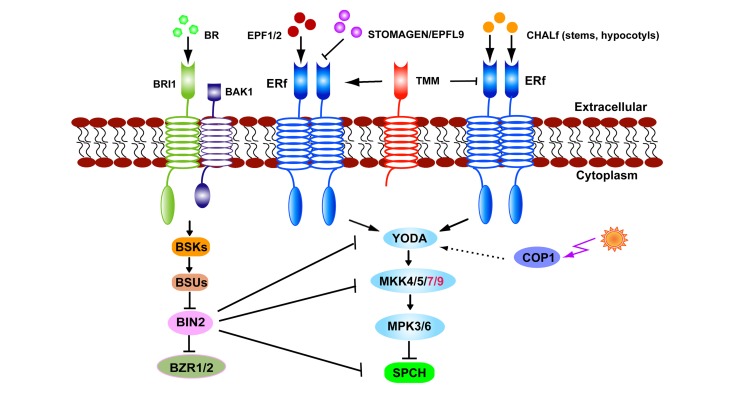
**The stomatal signaling pathway.** EPF1, EPF2, and STOMAGEN/EPFL9 signals are received by TMM–ERf leading to transduction and the YODA–MKK–MPK cascade. Light regulates stomatal production via COP1 which is upstream of YODA. In stems or hypocotyls TMM dampens CHALf peptide binding with the ERf receptor. BR signals received by BRI1–BAK1 inhibit BIN2 activity. BIN2 suppresses SPCH by directly phosphorylating or indirectly thorough YODA or MKK4/5.

While the *TMM* gene was named based upon the loss of function phenotype of excessive stomata in leaves, stomata are absent from stems and hypocotyls (Yang and Sack, 1995; Geisler et al., 1998; Bhave et al., 2009). The loss-of-function of *CHALLAH* (*CHAL*), which encodes the EPFL6 peptide from the EPF/EPFL family, has been shown to restore stomata to *tmm-1* stems and hypocotyls (Abrash and Bergmann, 2010). Two CHAL paralogs, *CHAL-LIKE1*/*EPFL5* and *CHAL-LIKE2/EPFL4*, are also involved in stomatal development (Abrash et al., 2011). Higher order mutations in *CHAL* family (*CHALf*) produce stomatal clusters in the hypocotyls of *tmm-1* mutants. A model has been proposed in which TMM dampens CHALf signaling while it promotes EPF1/2-ERf-mediated stomatal formation. When *TMM* function is lost, CHALf ligands inhibit stomatal initiation and differentiation via ERf receptors (Abrash et al., 2011; **Figure 2**). In contrast to the specificity of EPF1/2 to the stomatal lineage, STOMAGEN and CHALf peptides are secreted from internal tissues, indicating that underlying cells are also involved in optimizing the stomatal formation and patterning (Abrash and Bergmann, 2010; Kondo et al., 2010; Sugano et al., 2010).

## INTRACELLULAR SIGNAL TRANSDUCTION PATHWAY

The signals received at the cell periphery, such as by TMM–ERf receptors, must in turn be transduced to act on nuclear or cytoplasmic targets. Mitogen-activated protein kinase (MAPK) cascades are candidates for intracellular signaling pathways that connect extrinsic signals to stomatal development. Loss-of-function mutations in the *MAPKK kinase YODA* (*YDA*) gene lead to the massive overproliferation of stomata in the epidermis. Normally, the two daughter cells of an asymmetric division exhibit distinct cell fates, those of a meristemoid and SLGC, and they eventually form a stoma and a pavement cell, respectively. But in a *yda* mutant, both progeny develop into stomata (Bergmann et al., 2004). MPK3 and MPK6 function downstream of YDA-MKKs during stomatal development (Wang et al., 2007). The functions of the MAPK cascade in stomatal development have been systematically examined using the targeted expression of constitutively active (CA) and dominant-negative (DN) kinase variants in the stomatal lineage. Together these results reveal that the MAPK signaling pathway functions during each stage of stomatal development from initiation to differentiation (Lampard et al., 2009).

The CONSTITUTIVE PHOTOMORPHOGENIC 1 (COP1) protein acts as an E3 ubiquitin ligase that transduces light signals perceived by photoreceptors. Loss-of-function mutants of *cop1* display stomatal clusters but this phenotype is suppressed by the expression of CA-YDA, consistent with the MAPK signaling pathway mediating light signals that regulate stomatal production. This signaling pathway is parallel to that of TMM, but upstream of the basic-helix–loop–helix transcription factor SPEECHLESS (SPCH; Kang et al., 2009; **Figure 2**).

SPCH, MUTE, and FAMA are key regulators that direct three successive stages of stomatal development (Ohashi-Ito and Bergmann, 2006; MacAlister et al., 2007; Pillitteri et al., 2007). SPCH is required for epidermal cells to acquire an MMC fate and to undergo asymmetric entry divisions. A strong allele *spch-1* is completely devoid of stomatal lineage cells. Overexpression of *SPCH* increases the number of asymmetric divisions and leads to extra stomata. *SPCH* overexpression can restore stomatal formation to *tmm* hypocotyls, consistent with SPCH acting downstream of TMM (Ohashi-Ito and Bergmann, 2006). The functions of SPCH during stomatal initiation required INDUCER OF CBF EXPRESSION 1 (ICE1)/SCREAM (SCRM), that regulates freezing tolerance, as well as SCRM2 (Kanaoka et al., 2008; **Figure 1**).

Disruption of brassinosteroid (BR) biosynthesis, perception, or signaling caused opposite effects on stomatal production in cotyledons and hypocotyls (Fuentes et al., 2012; Gudesblat et al., 2012a; Kim et al., 2012; Khan et al., 2013). The serine/threonine glycogen synthase kinase 3 (GSK3)/SHAGGY-like BRASSINOSTEROID INSENSITIVE 2 (BIN2) phosphorylates YDA *in vitro* as well as the substrates of YDA, MKK4, and MKK5 (Kim et al., 2012; Khan et al., 2013). SPCH activity is inhibited after its being phosphorylated by MPK3 or MPK6 (Lampard et al., 2008). Thus the lowered MPK3/6 activity in BR mutants assumed the formation of excessive stomata in cotyledons (Kim et al., 2012). By contrast, BRs promote stomatal production in hypocotyls where BIN2 might directly phosphorylates SPCH residues that overlap with those targeted by the MAPKs, as well as BIN2-specific residues outside the MPK target domain (Gudesblat et al., 2012b). Either MPK- or BIN2-mediated phosphorylation leads to the degradation of SPCH protein and blocks entry into the stomatal cell lineage (**Figure 2**).

Since a similar organ-dependent stomatal phenotype is present in the *tmm* mutants (Geisler et al., 1998), a model about BR organ-specific effects was proposed (Casson and Hetherington, 2012; Serna, 2013). In cotyledons, BIN2 phosphorylates YDA or MKKs at low BR levels, and switches off the degradation of SPCH by MPKs, resulting in the production of extra stomata. In hypocotyls, the presence of CHALf signaling might lead to a reduction in MAPK activity. Consequently, the BIN2-mediated direct phosphorylation of SPCH is the predominant pathway in hypocotyls (Serna, 2013). Interestingly, BRs can induce new meristemoids to form in *tmm* hypocotyls, a phenotype similar to the presence of increased meristemoids in *tmm chal* hypocotyls (Fuentes et al., 2012; Gudesblat et al., 2012b), indicating that organ-specific functions of *CHALf* might be responsible for the opposite effects of BRs on stomatal production (**Figure 2**).

Detailed analyses of stomatal development in sterol biosynthesis *fk* mutants suggest that sterols (BR-independent) are required for stomatal cell fate determination and maintenance. Physically asymmetric divisions progress normally in *fk* mutants, but their cell-fate asymmetry is disrupted (Qian et al., 2013).

Additional plant hormones, such as gibberellins (GAs) can contribute to organ-specific effects of BRs. Stomatal formation in hypocotyls, but not in cotyledons, can be induced by GA or ethylene, and this effect is pronounced when both hormones are present. Conversely, no stomata form in hypocotyls of the GA-deficient mutant *ga1-3* (Saibo et al., 2003).

Abscisic acid (ABA) not only induces stomatal closure, but also prevents stomatal initiation, since stomatal numbers increase in the ABA-deficient *aba2-2* mutant. Time-course analysis reveals that meristemoid formation is prolonged in *aba2-2*. By contrast, in the ABA-over-accumulating mutant *cyp707a1 a3*, meristemoid formation is restricted. Compared to the wild-type, *SPCH* and *MUTE* transcripts are abundant in the *aba2-2* mutant but reduced in the *cyp707a a3* mutant (Tanaka et al., 2013). Interestingly, new *tmm* alleles display differential sensitivity to ABA in seedling growth and seed germination, but not in stomatal development (Yan et al., 2014).

Auxin widely regulates plant development by coordinating the placement and patterning of organs and cells. Dynamic changes of auxin activity during stomatal development were monitored using auxin input (35S::DII-VENUS) and output (DR5::VENUS) markers by time-lapse imaging. The disruption of auxin eﬄux induced a delayed switching from meristemoids to GMCs, indicating that auxin depletion is essential for M-GMC differentiation. The disruption of auxin eﬄux also causes excessive stomata to arise in clusters, indicating that auxin is also involved in stomatal stem cell fate determination (Le et al., 2014).

MicroRNAs (miRNAs) play important roles in regulating gene expression in multicellular plants and animals. The miR824 regulates the asymmetric division of SLGCs by repressing the *AGAMOUS-LIKE16 (AGL16*) gene in the stomatal lineage (Kutter et al., 2007). The components of the miRNA pathway *HYPONASTIC LEAVES1* (*HYL1*), *ARGONAUTE1* (*AGO1*), and the *HUA ENHANCER1* (*HEN1*) genes participate in stomatal production (Jover-Gil et al., 2012). Time-lapse analysis revealed that *AGO1* acts as a negative regulator in restricting the asymmetric spacing divisions in SLGCs. *AGO1* may act by negatively regulating *SPCH* transcript levels downstream of *TMM* (Yang et al., 2014a; **Figure 1**).

## REGULATION OF CELL DIVISION IN STOMATAL DEVELOPMENT

*Arabidopsis* stomata are generated after at least one asymmetric and one symmetric division. Thus division polarity is important for the regulation of cell fate determination, proliferation, and patterning during stomatal development. The BREAKING OF ASYMMETRY IN THE STOMATAL LINEAGE (BASL) and POLAR proteins are novel proteins regulating stomatal divisions (Dong et al., 2009; Pillitteri et al., 2011). The localization and levels of BASL and SPCH have been tracked in developing leaves which led to a “polarity switching” model that predicts the sitting of the BASL protein during successive divisions (Robinson et al., 2011). The regulation of asymmetric divisions during stomatal development has been comprehensively discussed in recent reviews (Lau and Bergmann, 2012; Pillitteri and Torii, 2012; Wengier and Bergmann, 2012). Here we emphasize recent work on the control of terminal divisions in stomatal development.

The loss of *FAMA* function induces cell overproliferation, resulting in the stacking of narrow epidermal cells that lack GC fate (Ohashi-Ito and Bergmann, 2006). The R2R3 MYB transcription factors *FOUR LIPS (FLP)* and *MYB88* function at the same stage as *FAMA*. *flp myb88* double mutants resemble *fama* mutants in that they harbor extra divisions, although the latter lack GCs (Lai et al., 2005). *CDKB1;1* is expressed specifically in the stomatal lineage cells. Reducing CDKB1 activity, either by overexpressing a DN form *CDKB1;1.N161*, or via the loss-of-function of both the *CDKB1;1* and *CDKB1;2* genes (*cdkb1;1 1;2*) blocks the symmetric division of GMCs, resulting in the formation of single GCs (SGCs; Boudolf et al., 2004; Xie et al., 2010). GCs usually harbor 2C DNA levels, but SGCs in *CDKB1;1.N161* have a 4C DNA content, consistent with an arrest during the cell cycle transition before G2-to-M. FLP can directly bind to a *cis*-regulatory element within the *CDKB1;1* promoter and can negatively regulate *CDKB1;1* transcript levels.

Chromatin immunoprecipitation microarray (ChIP-chip) analysis also reveals that many core cell cycle genes are putative transcriptional targets of FLP/MYB88, including *CDKA;1, CDC6, CYCD4;1* (Xie et al., 2010). The loss of *CDKA* function in *cdka;1* homozygous mutants, also results in SGCs forming in the epidermis. But SGCs in *cdka;1* mutants contain a 2C levels of DNA, indicating that *CDKA;1* acts at the G1-to-S transition of the cell cycle. Moreover, *CDKA;1*, like *CDKB1;1*, is also a direct target of FLP/MYB88 through binding to *cis*-regulatory elements in these promoters (Yang et al., 2014b). CDKA;1 activity is generally more important for the G1-to-S transition, while CDKB1’s are required for the G2-to-M progression, but the overexpression of *CDKA;1* can partially rescue GMC divisions in a *cdkb1;1 1;2* double mutant, suggesting that elevating CDKA;1 activity can at least partially substitute for CDKB1 activity (Weimer et al., 2012; Yang et al., 2014b). The combined loss of FLP/MYB88 and CDKB1 function, such as in the *flp-1 myb88 cdkb1;1 1;2* quadruple mutant, induces SGCs to undergo endoreduplication, that can lead to mean DNA levels of 6C in SGCs. Thus FLP/MYB88 can also conditionally restrict the G1/S transition (Lee et al., 2013).

Since CDK activation depends on its association with cyclin partners, the co-expression of *CDKB1;1* and *CYCLIN A2;3* (*CYCA2;3*) enhanced the kinase activity of CDKB1;1 and triggered ectopic cell divisions (Boudolf et al., 2009). Defective GMC divisions are present in *cyca2* mutants, while the *cdkb1;1 cyca2;234* quadruple mutant displays more SGCs than the *cyca2;234* triple mutant, suggesting that *CYCA2s* and *CDKB1s* synergistically promote GMC division (Vanneste et al., 2011). The overexpression of *CYCA2;3* at the stage when *FAMA* is expressed induced a differential increase in *CDKB1;1* expression in some subdivided GCs. Strikingly, ectopic *TMM* expression was present in some of these subdivided cells, indicating a cell fate reversion from a GC to a precursor cell fate (Yang et al., 2014b).

FAMA overlaps in function with FLP/MYB88 in limiting GMC division, but likely acts in a parallel or different pathway (Ohashi-Ito and Bergmann, 2006). However, the FAMA protein, like that of FLP/MYB88, also bind to *CDKB1;1* promoter (Hachez et al., 2011). Recently, a functional redundancy between FLP/MYBB and FAMA in maintaining the GC fate and integrity has been found (Lee et al., 2014). While a *FAMA* transgene driven by its native promoter, i.e., *proFAMA:cFAMA-GFP*, rescued the *fama* mutant phenotype of tumor-like clusters, over time this transformation generated a gain-of-function phenotype, that of the asymmetric division of GCs themselves. This subdivision produces two cells with unequal size and fate with the smaller daughter cell often developing into a stoma, leading to a “stoma-in-stoma” (SIS) phenotype. Notably, the levels of trimethylation on lysine27 histone3 (H3K27me3) of stomatal stem cell genes was disrupted, i.e., on *SPCH*, *MUTE*, and *FAMA.* The constitutive expression of the Polycomb-group gene *CURLY LEAF* was found to suppress this SIS phenotype. Moreover, a FLP transgene also induced a SIS phenotype (Lee et al., 2014). RETINOBLASTOMA-RELATED (RBR), is a homolog of the human tumor suppressor *Retinoblastoma* gene, which is involved in H3K27me3-mediated gene silencing (Gutzat et al., 2012). Down-regulation of *RBR* by RNAi induced GC subdivisions as well as the resetting of GC fate to that of stomatal lineage stem cells (Borghi et al., 2010; Lee et al., 2014). Both FLP and FAMA directly bind to RBR, suggesting that FAMA and/or FLP/MYB88 might interact with RBR in repressing stomatal stem cell genes.

Suppression of RBR in the leaf epidermis also leads to the formation of small cells, consistent with RBR also regulating asymmetric divisions in the epidermis (Desvoyes et al., 2006; Borghi et al., 2010). The RBR protein is phosphorylated predominantly by CDK kinases, such as CDKA;1 and CDKB1s (Nowack et al., 2012). Consistent with the role of CDK in stomatal cell fate determination, the loss-of-function in a *cdkb1;1 1;2* double mutant, in a *35Spro:CDKB1;1.N161* line, as well as in *cdka;1* mutants all lead to a reduced stomatal production (Boudolf et al., 2004; Xie et al., 2010; Yang et al., 2014a). The expression of *CDKB1;1* under control of the *CDKA;1* native promoter partially rescued stomatal formation in *cdka;1* mutants. A mechanism in which asymmetric and symmetric divisions are controlled by the CDK activity levels is presented (Weimer et al., 2012).

## CHALLENGES AND PERSPECTIVES

A key breakthrough in understanding stomatal development was the finding of a set of transcription factors required for successive stages of development that include lineage initiation, differentiation, and proliferation. Recent work demonstrates that the stability of the SPCH protein is regulated by multiple kinases. A remaining challenge is to define how SPCH proteins are selectively phosphorylated by different kinases *in planta* depending on different cells, organs, and growth conditions.

*SPCH* is proposed to be transcriptionally regulated by CDKA;1 via the regulation of RBR activity (Weimer et al., 2012). It is also possible that FAMA or FLP maintain GC fate by interacting with RBR to suppress *SPCH* expression (Lee et al., 2014). Future studies in different organs and cell types in response to signaling should help elucidate the precise spatial control mechanism of stomatal cell fate determination and maintenance.

## Conflict of Interest Statement

The authors declare that the research was conducted in the absence of any commercial or financial relationships that could be construed as a potential conflict of interest.
